# Esophagitis Dissecans Superficialis: A Case Report

**DOI:** 10.7759/cureus.44372

**Published:** 2023-08-30

**Authors:** Eli A Zaher, Parth Patel, Daria Zaher

**Affiliations:** 1 Department of Internal Medicine, Ascension Saint Joseph Hospital, Chicago, USA; 2 Department of Internal Medicine, University Clinical Hospital in Białystok, Białystok, POL

**Keywords:** proton pump inhibitors (ppis), parakeratosis, esophagitis dissecans superficialis, weight loss, endoscopy

## Abstract

Esophagitis dissecans superficialis (EDS) is a rare esophageal lesion characterized by sloughing of the esophageal mucosa. Typically asymptomatic and histopathologically nonspecific, diagnosis relies on endoscopic appearance. We report a case of an 81-year-old female who presented with an 8-pound weight loss in two weeks. Upper endoscopy showed severe mucosal changes with sloughing in the lower esophagus, consistent with EDS. Histopathology confirmed the diagnosis. No offending agents were identified, and high-dose proton pump inhibitors (PPIs) were initiated, resulting in symptom improvement. EDS remains poorly understood; it is associated with medication use, esophageal motility disorders, and autoimmune conditions. EDS should be considered in unexplained weight loss cases, with treatment focused on the discontinuation of culprits and PPI therapy.

## Introduction

Esophagitis dissecans superficialis (EDS) is a rare esophageal lesion characterized by sloughing of the esophageal mucosa. Diagnosis relies on typical endoscopic appearance as it is frequently asymptomatic and histopathologically nonspecific. Despite its seemingly striking appearance, complete healing without complications is frequently achieved following the withdrawal of offending agents. It mostly affects those of older age on multiple medications, although the mechanism remains unclear [1,2]. We present a case of an 81-year-old female diagnosed with EDS following an acute 8-pound weight loss.

## Case presentation

**Figure 1:**
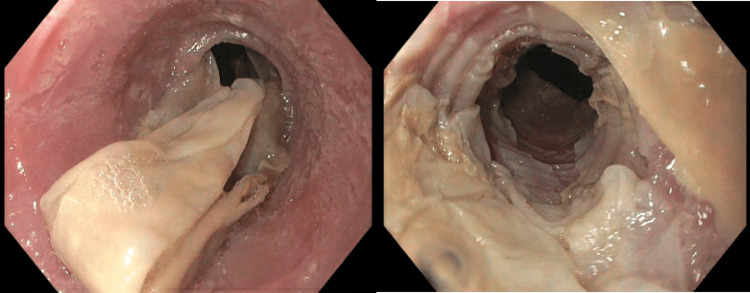
Upper endoscopy of the lower esophagus Upper endoscopy showing thin white folds of peeled mucosa consistent with diffuse severe mucosal sloughing.

An 81-year-old female with a history significant for hypertension, diabetes type 2, and hypothyroidism was admitted for episodic dizziness, generalized weakness, poor appetite, and unintentional weight loss of 8 pounds over two weeks. Workup for her dizziness was negative, although computed tomography (CT) of her abdomen and pelvis demonstrated incidental esophageal wall thickening with prominence of the gastric folds. Gastroenterology was consulted, and an upper endoscopy was performed to investigate the CT findings as well as her weight loss, which showed diffuse and severe mucosal changes characterized by sloughing in the lower third of the esophagus, suggestive of esophageal dissecans superficialis (Figure 1).

**Figure 2:**
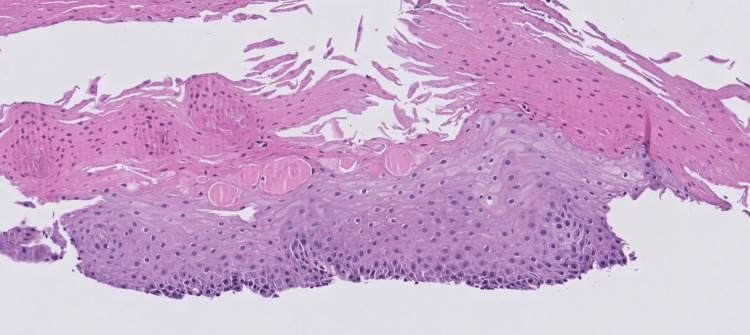
Hematoxylin and eosin staining of the distal esophageal mucosa Histopathology demonstrating hyperkeratosis, parakeratosis, and coagulative necrosis.

Histopathology of the mid and distal esophageal mucosa demonstrated squamous cells with parakeratosis, hyperkeratosis, and coagulative necrosis (Figure 2), which was compatible with the endoscopic findings.

Extensive history of the use of potential offending medications, toxins, and hot beverages was negative. She was thus started on high-dose proton pump inhibitors (PPIs) with gradual advancement of diet. Her symptoms improved thereafter with good tolerance to general diet and PPIs. She was discharged with instructions to continue on high-dose PPIs until a repeat upper endoscopy in one month; however, she was lost to follow-up.

## Discussion

EDS, also known as sloughing esophagitis, is a benign yet rare pathological finding with less than 100 cases described in the literature. It distinctly appears as columns of peeling mucosa with normal underlying esophageal tissue on upper endoscopy [1]. Histopathology further reveals parakeratosis and intraepithelial splitting, although it is nonspecific and can be misinterpreted as *Candida* esophagitis [2]. Additionally, clinical features such as odynophagia and dysphagia, and endoscopic features such as white patches also confuse EDS with *Candida* esophagitis. Despite these clear characteristics, the pathogenesis of EDS remains unclear, with the majority of cases considered idiopathic. One mechanism is believed to involve esophageal damage either iatrogenically or from exposure to hot beverages, smoking, toxins, and mediastinal radiation. Medications related to pill esophagitis such as iron tablets, potassium chloride, bisphosphonates, nonsteroidal anti-inflammatory drugs, antiepileptics, and selective serotonin reuptake inhibitors may lead to defective mucosal adhesion. Esophageal motility disorders and gastroesophageal reflux disease can also cause EDS from chronic retention and irritation. Lastly, autoimmune disorders, including celiac disease and pemphigus vulgaris, have also been associated with EDS [3-5]. It is more common in the elderly, especially if comorbid or taking five or more medications [6].

Clinical manifestations are broad, ranging from a silent incidental finding to odynophagia and hematemesis, although it is generally vague and modest [3-7]. Some may cough out pieces of the sloughed mucosa [2]. Our patient was admitted with poor appetite and an unintentional 8-pound weight loss for two weeks, a fairly acute and aggressive presentation.

Diagnosis relies heavily on endoscopic findings given the low specificity of histology and symptoms. It requires the identification of strips of sloughed mucosa longer than 2 cm in length with normal underlying tissue and without any adjacent ulcerations or friability. Severe cases appear as diffuse thin white membranes lifting off the esophageal epithelium, which may demonstrate extensive esophagitis and bleeding. The endoscopic changes in our case would be described as severe given the diffuse sloughing in the lower esophagus. Although histological analysis is nonspecific, it serves as additional support to the diagnosis. Prominent parakeratosis is typically found along with fragments of non-inflamed, necrotic epithelium. The basal layer should be intact and non-inflamed. Importantly, the sample may be contaminated by bacteria and fungi, so unnecessary antimicrobial treatment should be avoided [2,8,9].

There is currently no standardized management for EDS. However, most cases resolve spontaneously or following the withdrawal of offending agents. Temporary high-dose PPIs for four weeks are widely used for EDS, although they are believed to only prevent further damage rather than treat the underlying process. Further PPI course is determined by follow-up EGD findings after four weeks. In those with an autoimmune component who failed PPIs, immunosuppression may be attempted. Complete healing of the mucosa is frequently achievable without any long-term sequelae [6].

## Conclusions

Our patient exhibited a rare and aggressive presentation of EDS, coinciding with the striking endoscopic finding. It is important to consider EDS as part of the differential in those presenting with unintentional weight loss, even acutely. Most cases are idiopathic and respond well to the discontinuation of offending agents and the initiation of high-dose PPI therapy. For those refractory or presenting with severe cases, a trial of steroids may prove helpful, especially if suspicion for an underlying autoimmune process is high.
